# Development and current use of parenteral nutrition in critical care – an opinion paper

**DOI:** 10.1186/s13054-014-0478-0

**Published:** 2014-08-08

**Authors:** Mette M Berger, Claude Pichard

**Affiliations:** Service de Médecine Intensive Adulte et Brûlés, Lausanne University Hospital (CHUV), 1011 Lausanne, Switzerland; Clinical Nutrition, Geneva University Hospital, Rue Gabrielle-Perret-Gentil 4, 1211, Geneva, 14 Switzerland

**Keywords:** ᅟ

## Abstract

Critically ill patients depend on artificial nutrition for the maintenance of their metabolic functions and lean body mass, as well as for limiting underfeeding-related complications. Current guidelines recommend enteral nutrition (EN), possibly within the first 48 hours, as the best way to provide the nutrients and prevent infections. EN may be difficult to realize or may be contraindicated in some patients, such as those presenting anatomic intestinal continuity problems or splanchnic ischemia. A series of contradictory trials regarding the best route and timing for feeding have left the medical community with great uncertainty regarding the place of parenteral nutrition (PN) in critically ill patients. Many of the deleterious effects attributed to PN result from inadequate indications, or from overfeeding. The latter is due firstly to the easier delivery of nutrients by PN compared with EN increasing the risk of overfeeding, and secondly to the use of approximate energy targets, generally based on predictive equations: these equations are static and inaccurate in about 70% of patients. Such high uncertainty about requirements compromises attempts at conducting nutrition trials without indirect calorimetry support because the results cannot be trusted; indeed, both underfeeding and overfeeding are equally deleterious. An individualized therapy is required. A pragmatic approach to feeding is proposed: at first to attempt EN whenever and as early as possible, then to use indirect calorimetry if available, and to monitor delivery and response to feeding, and finally to consider the option of combining EN with PN in case of insufficient EN from day 4 onwards.

## Introduction

Critical illness requiring vital organ support is generally associated with an intense inflammatory response and requires bed rest, both factors favoring lean body mass catabolism. These alterations promote the risk of malnutrition, or aggravate a pre-existing malnutrition, and cause a related increased morbidity and mortality [1]. Critically ill patients depend on artificial nutrition for the maintenance of their metabolic functions and limitation of the underfeeding related to complications. Delivering adequate amounts of nutrients and energy should therefore be a basal preoccupation of the intensivist, like hydration and pain control. Giving your patient a FAST HUG (feeding, analgesia, sedation, thromboembolic prophylaxis, head-of-bed elevation, stress ulcer prophylaxis and glycemic control) is exactly this provision of basal care [2].

Current guidelines recommend early enteral nutrition (EN) as the best way to provide the nutrients [3,4]. Nevertheless, EN is a technique associated with practical problems worldwide, resulting in frequent insufficient feed delivery [5]. Further, EN may be contraindicated in some patients, such as those presenting anatomic intestinal discontinuity or splanchnic ischemia; hard contraindications are not very frequent in our experience, varying between 5 and 7% in the Geneva and Lausanne ICUs. Intravenous administration of macronutrients and micronutrients, called parenteral nutrition (PN), becomes recommended under certain circumstances (Table 1) [6] and is technically easier to deliver than EN. PN has therefore been overused in many ICUs, and has been associated with both metabolic and infectious complications [7]. In 2013 the medical community is left with great uncertainty regarding the place of PN in critically ill patients.

**Table 1:** **Indications for parenteral nutrition**

• Prolonged ileus >3 days	ᅟ
Mechanical obstruction	ᅟ
Generalized peritonitis	ᅟ
Peritoneal carcinosis	ᅟ
Abdominal distension on enteral nutrition	ᅟ
• Short bowel syndrome	ᅟ
Mesenteric infarction	ᅟ
Extensive small bowel resection leaving <1.5 m	ᅟ
• Severe malabsorption	ᅟ
Radiation injury to the intestine	ᅟ
High output fistulae (jejunal > ileal)	ᅟ
Inflammatory bowel diseases in acute phase	ᅟ
Splanchnic ischemia	ᅟ
• Time to reach full enteral nutrition or oral >5 days	ᅟ
• Insufficient energy intakes	ᅟ
• Hyperemesis gravidarum	ᅟ
• High risk of aspiration	ᅟ

This review aims at summarizing some critical metabolic changes in critically ill patients, and at explaining the historical background of PN development that impacts on its actual strengths and weaknesses.

## Impact of disease on lean body mass

Critical illness elicits a cascade of inflammatory, immune, endocrine and metabolic responses [8]. Critically ill patients are generally considered to be hypermetabolic, with exacerbated lipolysis, proteolysis and extracellular water gain associated with fluid resuscitation [9]. In response to inflammatory mediators and oxidative stress, and particularly in patients remaining acutely ill beyond the first 72 hours, proteolysis increases massively in excess of protein synthesis, a condition called catabolism that causes a rapid loss of lean body mass, mostly controlled by the ubiquitin–proteasome pathway [10].

**Figure 1:**
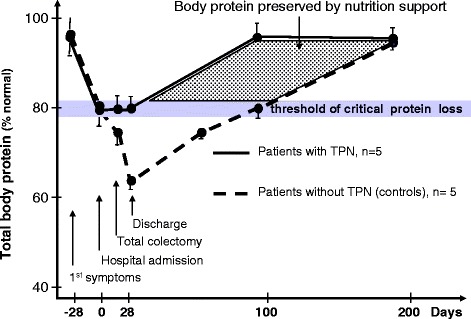
**Effect of nutrition support on total body protein in patients with an acute flare of ulcerative colitis.** Conversely to patients with standard care (broken line; mean age 47.6 ± 12.1 years, mean weight 59.4 ± 12.5 kg), ulcerative colitis patients with total parenteral nutrition (TPN; solid black line; mean age 42.6 ± 10.2 years, mean weight 60.8 ± 10.6 kg) received TPN from hospital admission until day 14 after surgery. Patients without TPN showed a rapid body protein loss that fell under the critical threshold before hospital admission, and worsened during the perioperative period. On the contrary, TPN prevented the worsening of protein body loss during the perioperative period and was associated with an earlier restoration (18 weeks earlier) of normal protein stores (mean ± standard deviation). Reproduced with permission from [14].

In the 1990s, using stable isotope techniques, it was shown that energy and protein requirements were higher in critically ill patients than in healthy subjects. Isocaloric PN was also shown to be well tolerated without generating undue hyperglycemia, but exceeding 1.3 g protein/kg/day intakes did not further increase protein accretion [11] (Figure 1). In critically ill trauma patients, PN providing 120% of the measured energy expenditure (EE) did not have any positive effect on protein metabolism, but only generated deleterious hypermetabolism [12]. Further, bed rest studies conducted by the European Space Agency demonstrated an intense protein catabolism related to physical immobilization [13], the latter being increased by hypocaloric feeding.

Critically ill patients combine both intense stress and physical immobilization [13] that may cause a rapid decrease of lean tissues, which in turn has an impact on respiratory and peripheral muscle function. After an acute disease, recovery of the muscle alterations is associated with an improved global functioning and quality of life [15]. The preservation of lean body mass appears particularly important for the final outcome. Optimal nutrition support during critical illness has been postulated to limit protein catabolism and promote faster recovery, although this concept has been challenged recently in a substudy of patients from the large Early Parenteral Nutrition Completing Enteral Nutrition in Adult Critically Ill Patients (EPaNIC) trial: PN administered from admission to the ICU as glucose followed by PN did not prevent lean body mass loss [16]. Contrasting with these results, an Australian study enrolling 1,372 patients with a short-term contraindication for EN showed clinical benefits in patients randomized to early PN with shorter mechanical ventilation and a better quality of life at 2-month follow-up; the parenteral intervention of course resulted in a better protein coverage compared with standard care [17]. More data on long-term outcome are needed.

## Historical development

**Figure 2:**
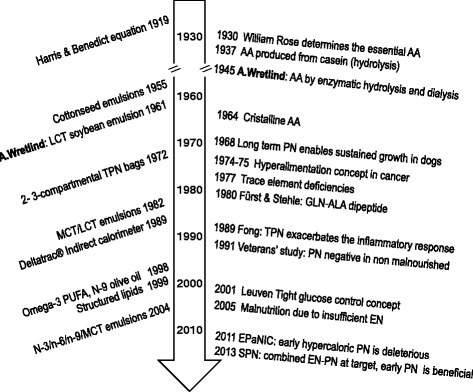
**Milestones in the development of artificial nutrition.** Development steps in energy requirements and lipid emulsions (left) and amino acid and glucose steps (right). Adapted with permission from [20].

Considering the development of PN is helpful in understanding the actual controversies. Figure 2 summarizes the major steps in the history of PN. Although attempts at feeding intravenously can be found as early as the 16th century, complete PN in its modern form was invented by Arvid Wretlind and colleagues in 1961 in Sweden [18] and in the USA [19]. From the start, this feeding technique saved many lives compromised by gastrointestinal failure, previously doomed to rapid death. Since its enthusiastic start in the 1960s, PN has evolved tremendously and has generated numerous contradictory publications.

Today, the production of metabolically balanced amino acid (AA) combinations remains a challenge. The development of the crystalline AAs [21] led to a better tolerance, but more prolonged PN treatments unmasked trace element deficiencies, requiring the development of balanced micronutrient solutions to compensate deficits resulting from the purification of the AA solutions.

The lipid emulsions remained the most difficult issue during decades. Severe complications of the cottonseed-derived lipid emulsions were observed in the USA, where their withdrawal led to incomplete PN (that is, without lipids) for decades. In Europe, however, thanks to the existence of the soybean solution using egg yolk phospholipids as an emulsifying agent, PN was complete (total PN) from the start. PN was widely used, often in patients without gastrointestinal failure, and despite the central venous access-associated complications. It is indeed much easier to deliver the prescribed amount of energy by the intravenous route than by the enteral route [22].

In the 1970s and 1980s the observation of devastating losses of lean body mass was the rationale for the delivery of hyperalimentation, as feeding was first called, using large glucose loads, erroneously supposed to suppress endogenous glucose production and thereby to prevent AA loss. The glucose–protein sparing strategy and the unavailability of lipid emulsions in the USA led to the prescription of high doses of dextrose exceeding the oxidizing capacity. Delivering >3,000 kcal/day was very common in the 1980s; hyperglycemia in the range 10 to 15 mmol/l was considered adaptive, and was generally not treated. The high glucose loads generated complications (excessive carbon dioxide production, respiratory failure, fever, additional metabolic stress, liver steatosis) [23]. It became obvious that parenteral hyperalimentation caused increased infectious complications compared with EN, which was frequently hypocaloric [24]. Only after the 2001 Leuven trial did the medical community become aware of the importance of controlling blood glucose levels by means of continuous insulin therapy [25], although the NICE SUGAR and Glucontrol trials subsequently demonstrated that intensive insulin therapy was potentially dangerous [26,27] and that less strict glucose targets compared with the initial normoglycemia (4.1 to 6 mmol/l) used by the Leuven team were safer.

Fong and colleagues showed that 5 days of PN in healthy subjects compared with EN exacerbated the inflammatory response to endotoxin [28]. In 1991 the Veterans study showed an absence of benefits and even deleterious effects of perioperative PN in patients without malnutrition [24]. These trials nearly destroyed the concept of PN, given that clinicians became very suspicious about potential deleterious consequences.

In the 1990s, technical advances in plastics enabled the development of double-compartmental and tri-compartmental bags in Europe, with separation of the macrosubstrates during storage, being easy to handle, and providing the necessary guaranties regarding stability and sterility of the solutions, reducing contamination, errors in prescription, and costs [29].

## Energy requirements

Determination of the patients’ energy and substrate requirements has proven much more difficult than expected. Evolving from the early phase of systematic hyperalimentation, the promotion of EN as the only correct way to feed patients has generated a second wave of ICU hypoalimentation from the 1990s that still persists [5,30,31]. A large proportion of severely ill patients leave the ICU with a cumulated deficit of up to 10,000 to 20,000 kcal [5,30,31], which roughly corresponds to about 5 to 10 kg lean tissues and 0.5 to 1 kg fat reserve. The guidelines have tried to promote a reasonable approach recommending 20 to 25 kcal/kg body weight in the early acute phase, to be increased to 25 to 30 kcal/kg in stabilized patients [6]. The aim was to initiate feeding early (that is, during the first 48 hours), preferentially by EN, to prevent the addition of an energy deficit to a previously malnourished patient or to avoid worsening the catabolic response of stress patients.

**Figure 3:**
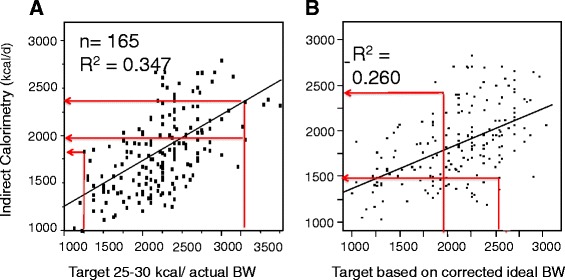
**Relationship between two commonly used equations and the value of energy expenditure.** Indirect calorimetry study on day 3 shows that both equations overestimated and underestimated energy expenditure in an unpredictable manner. **(A)** Pre-enrollment target 25 to 30 kcal/kg actual body weight (BW): arrows show the relation between the calculated energy target used for enrollment (25 or 30 kcal/kg/day) and the measured energy expenditure that became the target used from day 4. **(B)** Target of the supplemental parenteral nutrition patients recalculated using an equation based on a corrected ideal body weight, age and gender [39], which was used in the Early Parenteral Nutrition Completing Enteral Nutrition in Adult Critically Ill Patients (EPaNIC) trial [40] (corrected ideal body weight, age and gender [34] + absolute maximal target of 2,880 kcal).

The problem with all energy predictive equations is that they fail in nearly 70% of patients [32,33], being totally unreliable in the obese but also at the other end of the spectrum in patients with low body mass index <18. This unreliability occurs because EE in critically ill patients is highly variable depending on the initial injury, severity of the disease, nutritional status, time after admission and treatments, and an unpredictable variation of the body weight to EE ratio [34]. Many proposed equations are based on static variables (sex, age, weight, height) while others include dynamic variables (fever, minute ventilation, heart rate) [35]. Indirect calorimetry is considered the gold standard for determining EE, enabling feeding to be adapted to the measured EE. Two equations have been derived from such measurements and perform better than others: the Faisy–Fagon equation for mechanically ventilated patients [36], and the Toronto equation for major burns [37]. Figure 3 shows two static equations that failed to provide adequate orientation on the real energy target in the Swiss supplemental parenteral nutrition (SPN) trial compared with indirect calorimetry [38].

The tight calorie control study (TICACOS) is among the rare ICU studies where EE has been systematically and repeatedly measured, being determined every second day [41]. Unfortunately, the prescription of energy did not integrate non-nutritional calories (that is, associated with glucose for drug administration or lipid from propofol) despite supervision by the research team, and resulted in systematic overfeeding in the intervention group, which is likely to explain the observed increasing incidence of pneumonia and prolonged ventilation. Despite these immediate ICU complications, the TICACOS also showed that a better energy coverage in the calorimetry group with a combined nutrition support resulted in a reduced length of hospital stay and hospital mortality, but the numbers were too low to allow for definitive conclusions, needing repetition and extension of the study. Recently, the SPN trial showed that this individualized energy supply obtained by EE measurement in 65% of patients was rewarding in terms of reduction of nosocomial infections in severely ill ICU patients requiring prolonged ICU treatment [38]. Grau and colleagues have shown that liver alterations (defined as cytolysis, cholestasis or a combination of both) during enteral or parenteral feeding occur frequently if energy delivery exceeds 27 kcal/kg/day, and add to those liver alterations caused by sepsis and multiple organ failure [42]; the difference becomes significant after day 11 in patients on PN, ED producing less alteration related to lower energy intakes.

## Hypocaloric feeding: a real option?

Overfeeding is a threat, so some authors have proposed prescribing hypocaloric feeding; that is, 70 to 80% of the calculated target [43]. This strategy has been mainly developed for obese patients, in whom all of the equations invariably fail; the Penn State equation adapted for obese patients being among the closest to measured EE [44]. By contrast in hemodynamic management, an underprescription of norepinephrine would not be a treatment option in case of an arterial hypotension. Similarly, we need to prescribe what is considered adequate and optimal requirement for the patient, especially because the use of industrial formula implies that delivering low amounts of calories further lowers the protein delivery – a minimum energy is required to be able to benefit from proteins [9]. Moreover the international surveys show that underfeeding remains a serious threat [5], while patients with body mass index <18 experience a maximal variability of energy delivery.

Recent research has focused on autophagy, which belongs to the healing process as removal of mitochondria damaged during the acute phase of sepsis. This mechanism is a two-edged sword, however, which may cause cell death [45]. Insulin is a well-documented inhibitor of autophagy [46]. Overfeeding leads to increased insulin requirements to achieve blood glucose control. In the EPaNIC trial [16], the patients in the early PN group needed nearly double the amount of insulin for this purpose, probably reflecting overfeeding that was associated with depressed autophagy. Figure 3B shows that the “Leuven” equation results in frequent excessive targets when EE is verified by indirect calorimetry. By contrast, feeding in the SPN trial was guided by calorimetry, and no such increase was observed with identical insulin requirements in both arms [38].

## From substrate provision to pharmaconutrition

### Amino acids

The first crystalline AAs were unbalanced, containing about 50% glycine of low biological value. Introducing tyrosine, cysteine–cystine and glutamine was technically difficult due to stability and solubility problems. Glutamine was finally solubilized under the dipeptide form glutamine–alanine by Fürst and Stehle [47]. The concept of conditionally essential AAs emerged in this period; these are AAs that are supplied by food and synthesized under normal conditions but which during critical illness become deficient because of insufficient supply and increased consumption. Isolation of various AAs led to the possibility of using them separately, potentially as drugs.

Glutamine, the most abundant free AA in the body, which constitutes over 60% of the muscle free AA pool [47], is one of these conditionally essential AAs; depletion has repeatedly been shown to occur in critically ill patients and is associated with poor prognosis [48]. Well-conducted repletion studies have shown that glutamine administration is beneficial if administered along with an optimized nutrition therapy, particularly by the parenteral route, improving glucose control and achieving reduction of both infectious complications and mortality [49-52]. But glutamine cannot be provided in standard PN for stability reasons, which increases the risk of deficiency.

Two recent large prospective randomized clinical trials brought confusion, the doses of glutamine and their timing largely explaining the negative results. The Scottish SIGNET trial enrolled 502 patients with gastrointestinal failure [53]. The patients were randomized to receive daily 20.2 g glutamine or 500 μg selenium, or both, versus placebo for up to 7 days. There was no overall effect of glutamine on new infections or on mortality, while selenium was associated with less infectious complications if delivered for longer than 5 days. Several shortcomings of the study, including a very short administration time and a one-size-fits-all prescription of the ready-to-use PN bags, resulted in the delivery of a very low glutamine dose (0.1 g/kg/day) for a very short period, far below international recommendations [6]. On the non-effect side, a small American prospective randomized clinical trial in 44 patients randomized to three groups receiving either isonitrogenous enteral nutrition or 0.5 g/kg/day glutamine by the intravenous or enteral route for 8 days showed no difference in antioxidant status or other markers of oxidative stress [54]; importantly, about one-third of the patients had normal baseline glutamine levels.

The REDOXS trial reports data for 1,223 patients receiving the highest doses so far used of glutamine (0.78 g/kg/day supplied as 0.35 g/kg intravenously + 30 g/day enterally), about twice the recommended doses, in patients with severe organ failure (93% of patients in shock state and 33% with renal failure) starting within the first 24 hours of admission independently of nutrition [55]; that is, earlier than any previous trial. In addition, the glumatine group suffered more organ failure which might explain on its own the higher mortality. Surprisingly, in a subset of patients with plasma determination, only 31% of patients presented with a low baseline glutamine level (<420 μmol/l) whereas 15% of these patient had supranormal plasma glutamine values at baseline. The latter finding has been shown to be associated with increased mortality [56]. Pharmacological doses of glutamine in unstable patients are therefore to be avoided.

### Lipids

Many of the early problems as well as the progress of PN were associated with the development of lipid emulsions [23]. Prolonged use of the glucose and AA-based formulations with no fat was associated with essential fatty acid deficiency [23]. As fatty acids are more energy dense (about 9 kcal/g) than both AAs (4 kcal/g) and glucose (3.7 kcal/g), they enable reducing both the fluid load (important in the ICU patient) and the osmolarity of the solutions, permitting peripheral administration in some patients.

Since the development of the long-chain triglyceride solution by Wretlind and colleagues, major developments have occurred (Figure 2). Pulmonary and hematological side effects were observed, as well as an enhanced oxidative stress causing peroxidation of the unsaturated fatty acids; the latter may cause cell death and worsening of organ failures [21]. One of the first options aiming at minimizing oxidative stress was to partially replace polyunsaturated fatty acids (PUFAs) with oils rich in medium-chain triglycerides derived from coconut oil, which are less prone to peroxidation. Another advantage of medium-chain triglycerides is that they require less carnitine for mitochondrial penetration than long-chain triglyceride solution and are metabolized more rapidly, but they increase in a reversible manner the production of ketone bodies [57].

Progressively, it became obvious that the various PUFAs have proper modulating effects on the inflammatory response [58]. Medium-chain triglycerides and monounsaturated fatty acids derived from olive oil are considered the most neutral, while the more recently developed n-3 PUFAs derived from fish oil exhibit anti-inflammatory properties [58]. The intravenous administration of n-3 PUFAs seems associated with clinical benefits [59] and quick physiological effects [60]; development is ongoing and has been reviewed recently [61].

## Is parenteral pharmaconutrition worth it?

These variable results have lead to questioning the cost-efficiency resulting from glutamine and n-3 PUFA administration. The most convincing evidence comes from recent large-scale medico-economic studies. A large recent Italian study in 60,000 patients from 200 Italian ICUs strongly supports the use of glutamine containing PN [62]; the costs of treatment were completely offset by savings made by shortening the ICU stay and lower antibiotic costs. Similarly regarding n-3 PUFAs, an analysis including 23 trials in 1,502 patients showed that n-3 PUFA inclusion in PN resulted in a reduction of infection complications (relative risk = 0.61) and of lengths of stay [63], both in the ICU (nearly 2 days) and in the hospital overall (3.3 days), reducing the costs. Finally, an economic analysis of the Australian early PN study showed that the strategy of cautious early PN reduces hospital costs [64]. Well-conducted nutrition therapy, despite including a modest investment, is cost-saving in the end.

## What is the optimal timing for parenteral nutrition introduction?

While the indications for total PN (delivery of glucose, proteins, fat, and micronutrients) are unchanged, much controversy has arisen regarding its timing. Guidelines have been understood in divergent ways: the European Society of Clinical Nutrition PN guidelines have been interpreted as ‘deliver PN after 2 days’ [6], while the American Society of Parenteral and Enteral Nutrition guidelines have been understood as ‘no feed before day 7’ [65].

The large, prospective, EPaNIC trial randomized 4,640 patients to early PN (2 days with glucose from day 0) followed by full PN versus late PN (day 8) after ICU admission, and concluded that early PN was harmful, with later ICU discharge and more complications (including infections), despite the application of a tight glycemic protocol [40]. The EPaNIC trial used PN in unselected patients [38]. This last characteristic is precisely one of the major pitfalls of the study; early PN in low-risk patients may be harmful [66], confirming the Veterans trial results [24]. McClave and colleagues pinpointed various limitations of the study, such as the hypercaloric and low nitrogen intakes and the over-representation of cardiovascular surgery patients, which reduce its external validity [66].

The Swiss SPN prospective randomized controlled trial enrolled 305 patients on day 3 of admission to the ICU: enrollment criteria were that patients received ≤60% of the calculated energy target from EN, were expected to stay for >5 days, and were expected to survive for >7 days [38]. All patients were given a chance to be fed enterally, only those failing to achieve 60% of target were randomized to receive, or not, supplemental PN on top of EN in order to cover their measured energy needs after day 3. Energy targets were set by indirect calorimetry after day 3 (Figure 3A) or, if not technically possible, continued as 25 and 30 kcal/kg actual body weight/day for women and men, respectively. The difference between the indirect calorimetry value and the equation target varied between −1,000 and +1,000 kcal/day. Figure 3 shows the relation between these measurements and two commonly used equations: the simple 25 to 30 kcal/kg body weight equation [6], and a more sophisticated equation based on the corrected ideal body weight, age and gender [39] used in the EPaNIC trial. Patients were randomly assigned to continue exclusive EN or to SPN (EN + PN). The primary outcome was the occurrence of nosocomial infection from the end of intervention (day 8) until the end of the follow-up (day 28). The intervention group had less nosocomial infections (*P* = 0.03), and a lower mean number of nosocomial infections and of antibiotic days per patient, resulting in more antibiotic-free days. The study concluded that individually optimized energy supplementation with SPN starting 4 days after ICU admission could reduce nosocomial infections. The study has some shortcomings that are summarized in a series of letters [67a-d], particularly the fact that infections during the intervention were not considered. But indeed the primary endpoint was infections after the intervention (5 days of full feeding). The difference became only significant after a few days, a time delay that appears to be normal for a metabolic intervention that requires 3 to 5 days to become measurable, distinguishing nutritional therapy from pharmacological interventions. Under conditions of insufficient EN, SPN might improve clinical outcome, if it adequately covers basal energy requirements, enabling a normal metabolic response.

Doig and colleagues enrolled 1,372 Australian patients with a temporary contraindication to EN in an early PN trial [68]. The patients were randomized within 24 hours of ICU admission to receive either standard care or early PN. Their conclusion is that ‘the provision of early PN to critically ill adults with relative contraindications to early EN, compared with standard care, did not result in a difference in day-60 mortality. The early PN strategy resulted in significantly fewer days of invasive ventilation but not significantly shorter ICU or hospital stays’ [68]. The deleterious effects observed in the EPaNIC trial were not observed in the early PN group, which supports the findings in the SPN trial [38]; indeed, the energy targets used in the Australian trial were modest, based on the Harris and Benedict equation, thereby reducing the risk of overfeeding.

Finally, 14% of patients are plagued by diarrhea as shown by a recent trial including 1,595 ICU patient-days. EN may contribute to development of diarrhea because the delivery of more than 60% of the energy expenditure almost doubled the risk of diarrhea. This suggests that in some patients the combination of EN and PN may be helpful, reducing the burden and the cost of managing diarrhea (manpower, investigations, treatment) [69].

## How to monitor artificial feeding and parenteral nutrition

The clinical follow-up should integrate a close monitoring of the metabolic and gastrointestinal tolerance with a bundle combining clinical observations, a daily glycemic profile and insulin requirements to watch for potential overload. Monitoring should also include a weekly laboratory workout and observations of the changes over time of these variables (Table 2).

**Table 2:** **Suggestions for a systematic weekly monitoring of metabolic response to feeding, with interpretation of the changes**

**Variable**	**Mon**	**Tue**	**Wed**	**Thu**	**Fri**	**Sat**	**Sun**	**Mon**	**Interpretation**
Energy balance (daily, accumulated)	X	X	X	X	X	X	X	X	Daily delivery >110% or <80% of prescription: act accordingly to ↓ or ↑ intake
									Cumulated energy target over 3 to 6 days: <−4,000 kcal, beware and increase feeding; <−8,000 (−100 kcal/kg), danger
Glucose	4	4	4	4	4	4	4	4	↑: suspect overfeeding or infection; →, continue as is; ↓, improving condition
Insulin/24 hours	X	X	X	X	X	X	X	X	
Triglycerides	1				1			1	↑: non-nutritional fat intake? Nutritional fat? Sepsis?
ASAT ALAT	1				1			1	↑: sepsis? Drug toxicity? Overfeeding? Watch for glucose. →, continue
Prealbumin	1							1	↑: decreased inflammation and improved protein accretion; ↓: worsening of inflammation or insufficient protein intakes
Albumin CRP	1							1	Provide information on level of inflammation and severity of disease
Weight (actual)	X				(X)			X	↑: fluid accumulation? ↓: loss of fluid and lean body mass
Se, zn	?								In at-risk patients (CRRT, intestinal fistulae, prolonged feeding)

CRP, C-reactive protein; CRRT, continuous renal replacement therapy; ?, on demand in patients considered at risk.

## Towards a pragmatic approach

These trials are showing us the way to a reasonable nutritional therapy. Our aim is still to counteract a massive catabolism, but all patients are not equally exposed to this risk. The critically ill patient is an individual to whom mean values apply poorly. Energy equations are simple to calculate, but constitute an inadequate tool; measuring the individual requirement is essential to avoid both underfeeding and overfeeding. Starting EN within the first 48 hours at a slow progressive rate (20 ml/hour even in the sickest) is beneficial, including for non-nutritional reasons such as keeping bowel motility and IgA secretion [4]. If the gut is not functional with a transient or permanent contraindication to EN, a precise PN with low targets covering only the basal resting EE requirements, measured or based on the crude Harris and Benedict equation, may be initiated after 2 to 3 days in those patients presenting malnutrition on admission [68]. On the other hand, early overfeeding is deleterious, and is a real risk with PN [40]. After day 3, if the EN does not cover the measured expenditure, then a combined intravenous and enteral approach may prevent larger energy deficits [38]; the tolerance level to energy deficit seems to be somewhere around a cumulated balance of −50 to −80 kcal/kg body weight from admission before development of complications related to underfeeding. The concept implies a monitoring of feeding delivery to closely guide nutrition therapy.

An unequivocal approach – that is, one opposing enteral and parenteral feeding – is inadequate in most ICU patients [70]. However, a pragmatic and reasonable attitude seems the better deal for the individual patient; while PN is simpler to deliver than EN, its metabolic consequences are more complicated to handle. The authors’ experience is that PN requires tight monitoring and dedicated resources such as dieticians in the ICU [71]. PN saves lives, but easily causes overfeeding, with its deleterious side effects. We have to work at identifying and measuring needs more precisely, but also observing the individual tolerance to enteral or parenteral feeding, in order to optimize the global care of patients.

## Abbreviations

AA: Amino acid
EE: Energy expenditure
EN: Enteral nutrition
EPaNIC: Early Parenteral Nutrition Completing Enteral Nutrition in Adult Critically Ill Patients
PN: Parenteral nutrition
PUFA: Polyunsaturated fatty acid
SPN: Supplemental parenteral nutrition
TICACOS: Tight Calorie Control Study
